# High Throughput Multi-Omics Approaches for Clinical Trial Evaluation and Drug Discovery

**DOI:** 10.3389/fimmu.2021.590742

**Published:** 2021-03-31

**Authors:** Jessica M. Zielinski, Jason J. Luke, Silvia Guglietta, Carsten Krieg

**Affiliations:** ^1^ Hollings Cancer Center, Medical University of South Carolina (MUSC), Charleston, SC, United States; ^2^ Hillman Cancer Center, Department of Medicine, Division of Hematology/Oncology, University of Pittsburgh Medical Center, Pittsburgh, PA, United States

**Keywords:** CyTOF/mass cytometry, Cite/REAP-seq, high-dimensional analysis, bioinformatics, machine learning, biomarker

## Abstract

High throughput single cell multi-omics platforms, such as mass cytometry (cytometry by time-of-flight; CyTOF), high dimensional imaging (>6 marker; Hyperion, MIBIscope, CODEX, MACSima) and the recently evolved genomic cytometry (Citeseq or REAPseq) have enabled unprecedented insights into many biological and clinical questions, such as hematopoiesis, transplantation, cancer, and autoimmunity. In synergy with constantly adapting new single-cell analysis approaches and subsequent accumulating big data collections from these platforms, whole atlases of cell types and cellular and sub-cellular interaction networks are created. These atlases build an ideal scientific discovery environment for reference and data mining approaches, which often times reveals new cellular disease networks. In this review we will discuss how combinations and fusions of different -omic workflows on a single cell level can be used to examine cellular phenotypes, immune effector functions, and even dynamic changes, such as metabolomic state of different cells in a sample or even in a defined tissue location. We will touch on how pre-print platforms help in optimization and reproducibility of workflows, as well as community outreach. We will also shortly discuss how leveraging single cell multi-omic approaches can be used to accelerate cellular biomarker discovery during clinical trials to predict response to therapy, follow responsive cell types, and define novel druggable target pathways. Single cell proteome approaches already have changed how we explore cellular mechanism in disease and during therapy. Current challenges in the field are how we share these disruptive technologies to the scientific communities while still including new approaches, such as genomic cytometry and single cell metabolomics.

## Introduction

Since the early days of cell biology scientists have been using optical instruments to identify cell types in homeostatic conditions and diseases. With the wide introduction of flow cytometry in the early 70-ies markers and subsequent cell types have evolved but it was only in the last decade that the introduction of single cell transcriptome sequencing, high dimensional cytometry and imaging cytometry started revolutionizing the way we interrogate biological samples.

Isolation of multiple types of molecules (DNA, RNA, or protein) from a single cell simultaneously, stands at the beginning of each approach and having standardized and validated protocols for single cell solutions is surely the foundation of all the herein described approaches. Utilizing single cell genome, methylome, chromatin accessibility, while RNA or protein from the same cell can be used to profile the transcriptome, and proteome, different single cell omics profiles alone or in combination can serve as building blocks to construct a multi-omics profile for the same cell.

In this review article we will recapitulate the highlights of each of these technologies, analysis pipelines and discuss their potential to revolutionize future sample analysis, clinical trial design and ultimately redefine clinical research.

## A New Era of Single Cell Data Generation

### Pioneering Flow Cytometry

The first particle separator using the flow cytometry technology was employed in 1965 at Los Alamos National Laboratory (1) for sorting particles with different volumes to meet the needs of Mack Fulwyler. Meanwhile, Len Herzenberg, who was interested in a machine that could sort living cells based on their fluorescence, applied the design of the Fulwyler particle separator to build the first fluorescence activated cell sorting (FACS) instrument at Stanford University in the late 1960s (see the video Inventing the Cell Sorter, Herzenberg Lab, https://www.youtube.com/watch?v=Ro8P3w9BPhg).

The HIV/AIDS pandemic in the 1980s then gave a dramatic impulse to the technology of counting specific cells, since it became clear that the quantification of peripheral blood CD4+ T cells was crucial to follow the course of the infection, and eventually for monitoring response to therapy (2). As a consequence, the development of flow cytometers that had to be easy-to-use in all clinical laboratories, mainly focused on the proteome, and helped to widely disseminate this technology. Nowadays, flow cytometry is a commonly used tool in the field of immunology to finely dissect the diverse phenotypic and functional properties of immune cells.

Decades of development have created very robust flow cytometers aimed to deliver data from thousands to millions of individual cells from tubes or multi-well plates at acquisition rates of tens of thousands of cells per minute and expanding from the proteome, over genes to reporters of transcription. In order to have a wide dynamic range, the systems are designed for optimal signal to noise ratios. Typically, fluorescence tagged antibodies as well as molecular sensors (such as Ca2+-flux), and genetically encoded reporters (GFP, tdTomatoe, RFP, etc.) can be detected. The main limitation of this technology lies in the amount of available dyes, lasers and comparable detectors. Currently, up to seven lasers with emission wavelengths from 325nm to 650nm are used and tunable lasers are becoming increasingly common. Nevertheless, the overlap in the emission spectra limits the number of detectable markers.

Flow cytometers use either photomultipliers or avalanche diodes to convert fluorochrome-emitted light into electrical impulse. The advent of advanced detectors, such as spectral flow analyzers, first introduced in 1979 (3), allows the acquisition of biological information over multiple channels/probes. Modern high parameter flow cytometers, like the BD Symphony, the Beckham Coulter CytoFLEX, or the Sony Spectral Cell Analyzer easily allow the measurement of 20+ parameters in a single sample at high throughput.

Prior to expert guided or automated analysis, data from flow cytometers needs rigorous pre-processing, which includes compensation and data normalization. To compensate the spectral overlap automated approaches using fluorescence beads and software solutions can be used. Normalization represents a more complex issue. Traditionally, standardization of flow cytometry data is difficult as flow cytometry settings change over time also in the same instrument, therefore creating batch effects when samples are analyzed days or even months apart. This issue is also caused by the fact that in flow cytometry samples cannot be acquired using a truly multiplexing approach, rather they can only be acquired sequentially over days or weeks if a plate reader is available. Therefore, curative naming and metadata are necessary to identify sources of batch effects. To date, several software solutions are available to normalize fluorescence values between data sets (4, 5). Magnetic gates on sub-population landmarks (6) are one of these solutions and consist in expert-identification of cell sub-populations with subsequent use of a software tool that automatically adjusts the gate on the identified populations. However, while this and all other available solutions are effective when minor shifts occur during acquisition, they might not be suitable in case of more substantial shifts occurring during time intensive acquisitions or when multiple instruments or data from multiple sites are used.

### Mass Cytometry: A Truly Multiplexing Single Cell Technology

Mass cytometry is a new hybrid technology employing principles of flow cytometry and mass spectrometry. Introduced in 2009 (7), mass cytometry (or Cytometry by Time-Of-Flight, CyTOF) has pioneered a new era of high-dimensional single-cell analysis, surpassing the limitations imposed by the spectral overlap in conventional flow cytometry (8, 9). The new concept of mass cytometry is the use of high purity, stable, rare earth metal isotopes coupled to a target-specific probe for single cell labeling. These probes are detected based on the metals’ mass/charge ratios by inductively-coupled plasma time of flight mass spectrometry (10). Among the advantages of this technology are the absence of spectral overlap, which allows to realistically measure over 40 markers on a single cell; absence of tissue auto-fluorescence; true multi-plexing capacity using barcoding matrices, which allow to run up to 100 samples in parallel without compromising dimensionality. To date, mass cytometry has not only been performed on leukocytes by using antibody-labeled probes but also on cell lines, bacteria, nanoparticles and beads (11–15). The core technology is rapidly developing along with bioinformatics and reagent chemistry, thereby creating a largely universal and extendable next generation platform for multiplexing high-dimensional single-cell cytometry applied to translational research, systems biology, and biomarker discovery.

**Figure 1:**
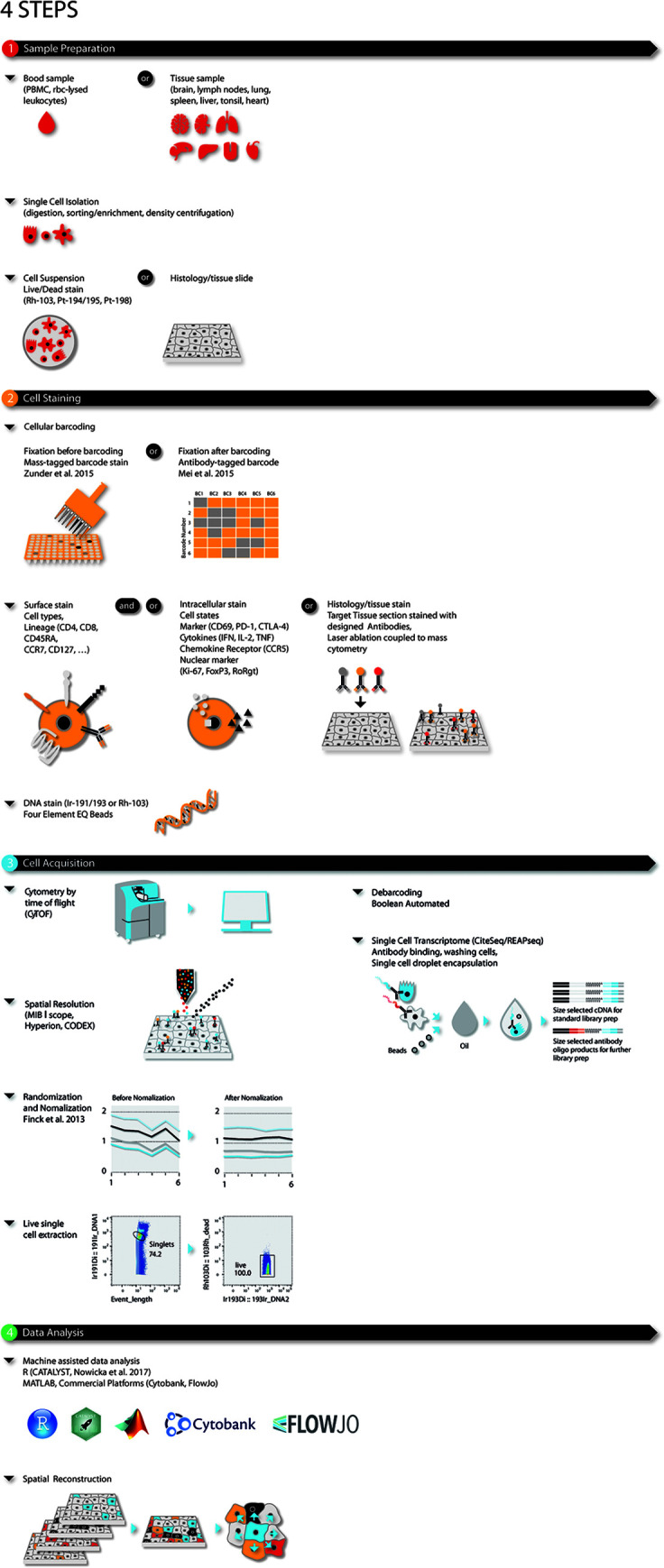
Four-step modular approach for high dimensional OMICS analysis for immune profiling during disease and (immuno)-therapy. Steps consist of (1) sample preparation, (2) cell barcoding and staining, (3) sample acquisition, and (4) data analysis. Briefly, single cells or histologic slides are prepared from blood, or fixed tissue samples. Single cells from blood or dissociated fresh tissues are barcoded, stained for live/dead, surface and/or intracellular markers and acquired using single cell solution mass cytometry. As an alternative CiteSeq can be utilized. Tissues on histologic slides are stained and acquired using imaging mass cytometry. For mass cytometry, data is bead-normalized and randomized. After de-barcoding data can be loaded in the bioinformatic analysis platform of choice.

Mass cytometry is ideally applied to research requiring high parametrization at single-cell resolution such as: (i) resolving cellular heterogeneity in complex mixtures of cells (e.g. bone marrow, blood or tissue); (ii) delineating complex phenotypes of isolated cell types, such as T-cell or myeloid subsets (16–19); or (iii) extracting maximum information out of limited (clinical) samples, such as tissue biopsies, blood samples from cancer patients and children (20–22). This latter makes mass cytometry ideal for large-scale immune monitoring and drug screening studies in clinical/translational research and systems immunology (**Figure 1**). The type of probes (antibodies) used by mass cytometry are the same as the ones used in flow cytometry with the caveat that mass cytometry is less sensitive than flow cytometry.

### Multiparametric Tissue Imaging

More recently, multiparametric (>6 markers) imaging using immunofluorescent or metal-labeled probes has been translated to tissues. Currently there are four commercially available systems: the Hyperion technology (Fluidigm), the MIBIscope system (Ionpath), the CO-Detection by indEXing (CODEX, Akoya) instrument platform and the MACSima (Miltenyi). The concept behind all of these technologies arose from Stanford University with the idea to extend high dimensional studies from solution mass cytometry to tissues, thus allowing efficient spatial resolution.

Mass spectrometry-based instruments like the Hyperion and MIBIScope vaporize histologic samples previously tagged with rare metals conjugated-antibodies and analyze their content in a mass spectrometer. The Hyperion system is an appendix to the Helios solution mass cytometer and can be operated using the same instrument with the addition of a laser ablation table to vaporize histologic samples (23). The MIBIScope is a stand-alone instrument, which uses an Ion beam for ablating rare metal stained tissues and needs special gold-coated slides for sample preparation (24, 25). The administered energy and speed of the ion beam can be regulated thus enabling different ablation speeds and tissue resolutions. Most importantly, the ion beam can reach sub-cellular resolution, therefore allowing the study of intracellular organelles and structures. The CODEX as well as the MACSima technologies use antibodies conjugated to unique oligonucleotide sequence barcodes. Target-specific barcodes with a dye-labeled reporter allow for highly specific detection.

All data generated on imaging platforms are displayed as data spots per area revealing the amount of probe that was bound to that spot when the tissue section was stained and ablated/screened. By plotting the data so that the single-spot data points are next to each other in the order they were originally sampled, highly multiplexed images of the tissue sections can be reconstructed. Together with the spatial information, whole tissues are electronically reassembled by using bioinformatics. By employing tissue microarrays and standardized staining panels, these technologies can be high throughput. Sensitivity for some probes on Hyperion is low. Due to a higher amount of energy transferred to the probe, the MIBIscope may offer higher sensitivity. As for the CODEX platform, this technology has just become commercially available and evaluation of performance in terms of sensitivity is still premature. Of note, the MIBIscope and CODEX systems are less tissue destructive, therefore allowing downstream use of the tissue for microdissection and further analyses. All technologies are relatively new to the broad scientific community and time and user preferences will clarify which technology is best to address each individual scientific question.

### Genomic Cytometry

Due to its long history and multiple validated analysis workflows, single cell (sc) RNA sequencing (scRNASeq) in combination with unsupervised machine-learning bioinformatics is nowadays the preferred approach to in-depth reveal the complexity of the cellular landscape in multiple diseases. In 2011 a pioneering study from Wigler and colleagues using scRNASeq showed that the metastatic dissemination is the consequence of a single clonal expansion (26). Increasing scRNASeq throughput (27) (28), has enabled the identification and characterization of novel or rare cell types (29), in addition to providing insights into the underlying mechanisms of cellular development (30) and response to therapeutic interventions (31).

However, proteins, not mRNAs, are the primary targets of drugs and protein abundance cannot necessarily be inferred directly from mRNA abundance (32–35). An unbiased view of proteins is thus necessary to model cellular dynamics and response to environmental and therapeutic perturbations. To address this need, recently, new cross over technologies using specific protein-targeted tags and untargeted transcriptomic or targeted genomic approaches have been developed. Cellular Indexing of Transcriptomes and Epitopes by Sequencing (CITE-seq) (36) and its sister technology RNA Expression and Protein Sequencing assay (REAP-seq) (37) use DNA-barcoded antibodies to convert detection of proteins into a quantitative, sequenceable readout. CITE-seq uses biotin-conjugated barcodes, while in the REAP-seq technology barcodes are directly bound to the probes. These antibody-bound oligos act as synthetic transcripts that are captured during most large-scale oligodT-based scRNA-seq library preparation protocols (e.g. 10x Genomics, Drop-seq, ddSeq).

## Biomarker Discovery and Novel Target Identification

### scRNA-Seq in Biomarker Discovery

According to the DNA-RNA dogma, DNA provides the code for RNA, which in turn is translated into protein (38). The majority of cell populations studies from complex heterogenous tissues such as cancer has been conducted on bulk samples, which read the average signal within a population thus preventing measurements of the single cell variation. The widespread knowledge of transcriptomic analysis fueled the study of single cell genes, transcriptomes and proteomes in several different diseases and cell types.

Non-invasive single cell-based sequencing of liquid biopsy has been proposed to screen circulating clonal population of cancer cells (39). For these reason, sophisticated methods to capture, enrich and sequence circulating tumor cells (CTCs) have been optimized. In metastatic breast cancer, scRNA analysis of CTCs has been performed using Hydro-Seq: high-efficiency-cell-capture-contamination free scRNA-seq. Using this powerful method, the authors revealed clinically relevant markers to identify CTCs and, interestingly, inter- and intra-patient transcriptome heterogeneity (40). In addition, the use of single cells sequencing enabled the identification of disease-associated cellular profile and interactome, which have been subsequently validated across 13 different diseases such as asthma, atherosclerosis, breast cancer, chronic lymphocytic leukemia (CLL), Crohn’s disease, eczema, obesity, influenza, psoriasis, seasonal allergic rhinitis, type 1 diabetes, tonsillitis and ulcerative colitis. This further supports the universality and the power of scRNA-seq for biomarkers detection and identification of therapeutic targets (41). The breakdown of immune cells activation and interaction have been analyzed by single cells studies during bacterial infection: over 7000 human PBMC *ex-vivo* infected with *Salmonella* have been sequenced using 10X Genomics with the aim to generate a detailed picture of infection-induced cell states (42).

A similar dynamic of the immune cells can be investigated in the tumor microenvironment. In hepatocellular carcinoma the combination of two scRNASeq technologies, SMART-Seq2 and drop-based platform, revealed six different macrophage clusters and a novel class of LAMP3+ mature dendritic cells, which dampens T cell antitumor functions. Additionally, the analysis of cell populations in multiple organs and body sites has revealed both the migration paths of immune cells and their origins (43). Single cell studies of lymphoid cells in cancer are also leading to the discovery of stronger predictors for disease and treatment outcomes. TCGA multi-omic data was collected across several cancers to identify novel markers from tumor infiltrating lymphocytes as key indicators for prognosis. Protein and mRNA expression profiles were correlated with survival curves leading to the discovery of *GPR18* as a better prognostic indicator over the previously established CD20+ (44). The interaction between immune cells and cancer can also be useful to identify novel therapeutic strategies. In this context, using scRNA-seq, lymphoid cells generated in the gut mucosa have been proposed as modulators in colorectal cancer. Various differentiation states can also play significant roles in the tumor microenvironment and be potentially relevant for novel immunotherapy strategies (45). In addition to the interaction among different immune cell subsets, scRNA-seq has proved valid to investigate the interaction between immune cells and non-immune cells such as tumor cells and stroma (46). The power of scRNA-seq in exploring cell heterogeneity within the same population has been also applied to the analysis of HIV permissiveness in CD4+ T cells. In this study CD298, CD63 and CD317 have been identified as a biomarker signature of cell permissiveness to the viral infection (47).

Besides study focusing on CTC and immune cells in the context of cancer, scRNA-seq has recently been used to unearth a range of clinically relevant non-immune markers from tumor samples. Recent work has defined cell populations within malignant osteosarcoma using scRNA-seq and Monocle 2 for pseudo-time analysis. Markers identified with this method were accurate in predicting metastases and disease recurrence (48). Similar techniques were applied to existing data to define novel biomarkers for hepatocellular carcinoma (49). Zhao et al. exposed glioblastoma samples to different chemotherapeutics and used scRNA-seq expression profiles to predict drug response in individual patients (50). A different study focused on glioma used existing multi-omic data, including scRNA-seq, to identify novel biomarkers in cerebrospinal fluid that can be used to assess diagnosis, prognosis, and directions for therapy (51). In patients with lung adenocarcinoma, scRNAseq on samples collected at different time points during disease, including at the time of metastases formation, enabled the identification of markers of cellular reprogramming and immunosuppression (52).

Lately, single cell methods have been employed to characterize SARS-CoV-2. Using scRNA-seq on COVID-19 positive patient bronchoalveolar lavage fluids (BAL), key immune cell populations were found to predict disease severity (53). In another study, peripheral blood mononuclear cells (PBMC) were collected from patients in the early recovery phase and using scRNA-sequencing Wen et al. showed a pro-inflammatory state following the primary infection, therefore suggesting that patients should be monitored for up to one week after the primary infection wanes (54). Using previously collected scRNA-seq data, one study developed a pipeline for identifying drug targets for COVID-19 and another combined this data with viral receptor interaction information to identify mechanisms of COVID entry across multiple organs (54, 55).

### Single Cell Proteomics in Biomarker Discovery

The Nolan laboratory has conducted pioneer work to adapt analysis of single cell proteomes to immunology using CyTOF. The more abundant proteomic information from each single cell enabled by mass cytometry provides a much broader landscape for different types of biological questions such as the frequency of immune cells in response to a stimulus (56) and the correlation between tumor pathology and phosphoproteins signaling (57). Relevant clinical information such as immune response during diseases (58, 59), assessment of clinical recovery after surgery (60), and guidance to effective therapy (22, 59) has also been obtained using this method. Over time, several research groups have combined this technology with clinical samples, clinical data and novel functional assays to assist easier disease diagnosis (61). Mass cytometry has been crucial in deciphering dendritic cell ontogeny (19, 62), B cell development (63) and in characterizing the human mucosal immune system in gastrointestinal pathologies (64). Krieg et al. used high-dimensional single-cell mass cytometry to characterize subsets of immune cells in the peripheral blood of metastatic melanoma patients before and during anti-PD-1 immunotherapy (20). Thirty surface markers were first assayed in the leukocytes and a set of T cell subsets at the different stages of differentiation and activation. Then, functional phenotypes of T cells and myeloid cells were extensively characterized with single-cell proteomic profiling to discover the difference between responders and non-responders to anti-PD-1 immunotherapy. This study showed for the first time that the frequency of CD14+ CD16− HLA-DR^hi^ monocytes may serve as a prognostic biomarker of progression-free and overall survival before immunotherapy (20). Wei et al. characterized single tumor-infiltrating T cells (TILs) with mass cytometry by assessing 33 surface and 10 intracellular markers, including non-T cell lineage markers, T cell differentiation and activation markers, and T cell lineage transcription factors. This study showed that both anti-CTLA-4 and anti-PD-1 antibodies expand exhausted-like CD8 T cells, and that anti-CTLA-4 antibody modulates an ICOS+ Th1-like CD4 effector subset for engaging exhausted-like CD8 T cells. Wei et al. discovered the distinct cellular mechanisms of antitumor immune responses induced by CTLA-4 and PD-1 blockade in the preclinical and clinical analysis (65). Recently, Spitzer et al. proposed that effective cancer immunotherapy depends on systemic immunity. To validate this hypothesis, they assessed antitumor immune response in lymph node, spleen, blood, and bone marrow in mouse models of breast cancer using 39 immune markers for surface and intracellular proteins. This study provided evidence that a population of CD4+ T cells in peripheral blood could predict systemic active immunity required for tumor rejection (66). In addition, Becher et al. showed circulating auto-reactive T cells in patients suffering from narcolepsy and multiple sclerosis (21, 67).

Compared to scRNAseq and fluid phase mass cytometry, imaging mass cytometry (IMC) is still in its infancy mainly because of the still limited distribution of the technology and expert laboratories across the world. To date, the most exhaustive results obtained using IMC have been produced by the Bodenmiller laboratory using the Hyperion system and focused on the characterization of immune contexture and tumor microenvironment in breast cancer (23, 68). We envisage a widespread dissemination of high dimensional imaging using the Hyperion and previously mentioned sister technologies. This in turn will contribute to the generation of very comprehensive atlases, which will be of great value for a deeper understanding of cellular interactions during health and disease.

## Integration Using Bioinformatcs

### Pre-print Platforms

One of the biggest challenges posed by single-cell-omic approaches is the simplification of data processing and analysis, so that workflows can become readily available not only for research purposes but also for clinical application. Several solutions for data storage and processing are nowadays free or commercially available *via* subscription or licensing.

Free platforms are FlowRepository (www.flowrepository.org), ImmPort (import.niaid.nih.gov), and ImmuneSpace (www.immunespace.org). One commercial service, **CytoBank**, offers a cloud-based network that allows easy accessibility to data and code in a user-friendly format. Online support for analysis or workflow problem solving is also available. CytoBank includes analysis packages for FlowSOM and CITE-seq, which are used for proteomic and transcriptomic data sets, respectively.

**FlowJo** and **SeqGe** are two additional large platforms from BD, which offer user-friendly workflows. Each of these applications uses extension plugins that can provide quality control, data analysis, and visualization tools. Because of the user-friendly data analysis, there are far more limitations on how the user controls the data meaning that the preprocessing of single cell data files requires higher refinement prior to using such a service and often may still require some expertise to ensure the analysis is robust and significant. Much of the preprocessing and detailed aspects of data organization are not controllable within such platforms, which means there is still room for mistakes.

**VDJViewer** was specifically developed with the intention to provide single cell data processing for users that do not have advanced bioinformatic expertise, therefore making immunologic single cell data more accessible (69). In addition to modeling antigen markers, scRNA seq, and meta data, this application can also perform pseudo-time analysis and dimensionality reduction (69). As more data and platforms like VDJViewer become available, there is also a need to improve the models that operate within a given platform.

While these platforms are undoubtedly allowing wet laboratories to successfully analyze complex -omic data sets with minimal assistance of trained bioinformaticians, they are often limited in their application especially when dealing with complex datasets.

An alternative approach to commercially available solutions is the use of preprint platforms, which rely on community feedback for rapid development of data processing solutions. Because there is no “universal standard method” to process single cell data from different experiments and combining these large data sets requires creative and strategic methodology, actively seeking user feedback can streamline solutions. The use of these platforms is free and only requires updated software and operation systems. Further, it ensures reproducibility, which is essential when the aim is the generation of a data cloud containing standardized cell and diseases atlases as references.

There are several leaders in preprint workflows, including **Bioconductor** and **F1000**. F1000 is a life sciences publisher with four primary divisions. F1000Research is the branch that focuses on data sharing. Bioconductor is a ready to download software package available within F1000Research, written in R and used as a bioinformatics community platform. Bioconductor offers free download of user-friendly workflows, sample data sets, and data analysis software packages. Additionally, an updated package is released every six months. Some of the common workflows within Bioconductor include scRNAseq and proteomic differential analysis. One example is Catalyst, a software program within Bioconductor developed by Mark Robinson laboratory. This program was specifically developed to offer a simplified way to analyze single cell mass cytometry data. One recent application of Catalyst allowed the identification of immunologic phenotypes pertinent to lung cancer prognosis (70). Other software programs within Bioconductor optimize single cell RNA-seq and epigenomic data. Among these softwares, the ChAMP software program recently allowed the identification of novel epigenomic biomarkers in colorectal cancer (71).

Another preprint leader, the **10X genomics** platform, is an organization focused on the development of single cell technologies and analysis pipelines. Although CellRanger is best used by commercially available kits, the free available software is gauged at the analysis of single cell data. Recent technologies within the 10X genomic project include the Visium Spatial Gene Expression Solution, which was developed for spatial resolution of transcriptomic data. In this assay, total mRNA samples are collected and processed by linking the transcriptomic barcode to a defined region of interest on the slide, thus providing spatially relevant information. In addition to technology development, 10X has led efforts to expand the power of single cell analysis. In 2017, 10X initiated a project in partnership with the Human Cell Atlas to create a single-cell based atlas available as a reference, which would initially include scRNA-seq data.

## Challenges

The field of high dimensional data poses several challenges, which in our view limit the accessibility of high dimensional technologies to clinical trial evaluation and drug discovery. The challenges are mostly related to three major areas: 1) data quality, 2) computational tools, and 3) training of the end user and generating the infrastructure.

### Challenge One—Data Quality

Examples of high dimensional data include genomic, transcriptomic, proteomic, and microbiome data, which are derived from different sources and are collected in a variety of distinct formats and often over several sites. This data is complemented by patient data/medical records. Errors occurring during measurement or during processing can compromise the reproducibility and the use of the generated data. To overcome this hurdle, details about data collection and generation must be transparently reported and each processing step must be documented to avoid or minimize data alteration. Additionally, wet laboratories must follow strict standards during sample collection, storage, processing and acquisition. It is also important that data collection, processing and management follow pre-defined national or international standards. The national FAIR (findable, accessible, interoperable, reusable) initiative is one attempt to imply such a standard (72). Notably, attempts in ensuring procedural standards must be supported by the application of ethical standards to protect the privacy of the participating patients (73). Furthermore, throughout processing and management data must remain reliable and therefore be complete, of high quality, diverse, relevant to the question asked, timeless and accurate (74). Furthermore, data quality must be maintained upon data compression, storage, transfer and analysis and the entire process must be reproducible.

### Challenge Two—Computational Tools

A desirable goal in the clinics is the creation of a FDA-approved software that can support clinical decisions (75). A prerequisite to this is the development of well tested wet lab protocols and computational tools, which can be easily used by users with a diverse knowledge level encompassing computational scientists, wet lab scientist and medical personnel (76). Open shared resources and code transparency as well as snapshots of program development can vastly enhance computational tool development and make the field more attractive to a wide group of users. Most importantly, wet lab protocols must be developed and pre-tested over several sites. Docker engines, which allow running software in a container, therefore making its installation independent on the environment, resulted in the use of software by a larger community (77). In this context, it will be of utmost importance having accessible, large and free data sets to allow for the testing and validation of software tools on real world data (78). Approaches such as the moonshot atlas initiative and the whole genome sequencing project are some of the examples.

### Challenge Three—Training of the End User and Generating the Infrastructure

With the continuous growth of the big data field, the training of wet lab and clinical scientists in computational sciences becomes essential (76). Ideally the training should be multi-disciplinary and cover a broad array of concepts such as molecular diagnosis, cellular immunology, computer programming, system biology and patient care. This broad rather than specialized knowledge will empower the next generation of students to use new software tools with ease. This can be done in specialized PhD programs or in the form of on job training degrees. Most importantly, to optimally achieve these objectives, hospitals and universities should provide the infrastructure, such as cloud computing, high speed internet connections in central as well as rural areas, easy access to the necessary software and sufficient support staff.

## Conclusion

There are endless challenges facing multi-omic data processing, as it is critical to consistently assess analysis methods and avoid over fitting. Part of this challenge is interpreting how the individual cell features and their interaction with other cell types contributes to specific outcomes and therefore designing appropriate algorithms. Such theory is relevant in economics and consumer behavior and many of these models have operated as examples for single cell analysis. For instance, economic game theory models weigh the net effects of individual decisions and small subgroups to understand what drives certain economic trends and this type of strategy has been used to create novel analysis methods for single cell data (79).

Furthermore, when dealing with multi-omic approaches layers of single cell data need to be stacked. Therefore, another challenge is the creation of robust systems to process individual data sets as well as effective strategies to combine multiple data sets and produce meaningful outcomes. Novel technologies like single cell proteomics, single cell sequencing, and single cell spatial resolution are rapidly developing. As more and more laboratories world-wide get access to these technologies, network like the Immuno-Oncology Translational Network (IOTN) develop reference atlases of tissues and lay the basis to analytical tools. With these atlases as reference we are witnessing the beginning of tremendous insight into immune and disease mechanisms during (immuno)therapy.

## Author Contributions

JZ and CK drafted the review. SG and JL gave feedback and performed editing. CK and SG finalized the review. All authors contributed to the article and approved the submitted version.

## Funding

Supported by Hollings Cancer Center startup funds (#87455) for CK; the Abney Foundation MD/PhD scholarship to JZ; ACS-IRG grant to SG.

## Conflict of Interest

The authors declare that the research was conducted in the absence of any commercial or financial relationships that could be construed as a potential conflict of interest.
